# Family history of cancer and renal cell cancer risk in Caucasians and African Americans

**DOI:** 10.1038/sj.bjc.6605680

**Published:** 2010-05-04

**Authors:** S Karami, K Schwartz, M P Purdue, F G Davis, J J Ruterbusch, S S Munuo, S Wacholder, B I Graubard, J S Colt, W-H Chow

**Affiliations:** 1 Division of Cancer Epidemiology and Genetics, National Cancer Institute, 6120 Executive Boulevard, MSC 7242, Bethesda, MD 20892-7242, USA; 2 Karmanos Cancer Institute, Departments of Family Medicine and Public Health Sciences and Population Studies and Prevention Program, Wayne State University, 110 E. Warren, Detroit, MI 48201, USA; 3 Division of Epidemiology and Biostatistics, School of Public Health, University of Illinois, 877 SPHPI M/C 923, 1603 W. Taylor Street, Chicago, IL 60612, USA; 4 Information Management Service Inc., 6110 Executive Blvd Suite no. 310, Rockville, MD 20852, USA

**Keywords:** renal cancer, kidney cancer, family history of cancer, race, Blacks

## Abstract

**Background:**

The association between renal cell carcinoma (RCC) risk and family history of cancer has not been examined with an adequate number of African Americans (AAs).

**Methods:**

In a population-based case–control study, unconditional logistic regression was used to calculate the association between RCC risk and a family history of cancer among 1217 RCC cases and 1235 controls.

**Results:**

Increased RCC risk was shown for subjects with at least one first-degree relative with kidney cancer (odds ratio=2.29; 95% confidence interval=1.31–4.00). No differences in risk were observed when analyses were stratified by race. For Caucasians, excess risk was observed among those reporting a sibling with kidney cancer, whereas for AAs, increased risk occurred among subjects reporting either a sibling or parent affected with the disease. A family history of non-renal cancers, and those related to smoking or to the von Hippel–Lindau syndrome, revealed no association with RCC risk.

**Conclusion:**

The RCC risk associated with a family history of kidney cancer is similar among Caucasians and AAs.

In the United States, malignant tumours of the kidney account for nearly 4% of cancer diagnoses and 2% of cancer deaths (Jemal *et al*, 2009). Adenocarcinoma of the renal parenchyma (renal cell carcinoma (RCC)) is the most common form, accounting for more than 85% of kidney cancers (Chow and Devesa, 2008). Since 1950, in the United States, there has been a 126% increase in RCC incidence (Paglino *et al*, 2007), with higher rates reported among African Americans (AAs) than among Caucasians (Chow and Devesa, 2008). The increasing rates of RCC over time may reflect, in part, the increasing use of imaging modalities (Paglino *et al*, 2007; Chow and Devesa, 2008; Patard, 2009). However, as the increase involves all tumour sizes, not just those at the local stage (Patard, 2009), imaging modalities alone do not entirely explain the increase.

The aetiology of RCC is complex, with both environmental and hereditary components suspected to have a role. Smoking, obesity, and hypertension are primary risk factors that may explain half of all RCC diagnoses in the United States (Benichou *et al*, 1998; Chow and Devesa, 2008). RCC risk has been examined in relation to familial history of cancer in a number of epidemiological studies (McLaughlin *et al*, 1984; Kreiger *et al*, 1993; Goldgar *et al*, 1994; Mellemgaard *et al*, 1994; Schlehofer *et al*, 1996; Gago-Dominguez *et al*, 2001; Czene and Hemminki, 2002, 2003; Gudbjartsson *et al*, 2002; Negri *et al*, 2006; Hung *et al*, 2007; Randi *et al*, 2007; Clague *et al*, 2009). Many of these studies have shown a positive association with a family history of cancer (Goldgar *et al*, 1994; Mellemgaard *et al*, 1994; Schlehofer *et al*, 1996; Gago-Dominguez *et al*, 2001), particularly when the affected relative is a sibling (Czene and Hemminki, 2002; Gudbjartsson *et al*, 2002; Negri *et al*, 2006; Hung *et al*, 2007; Clague *et al*, 2009).

Despite the racial disparity in RCC incidence, the numbers of AAs studied have been insufficient to examine their risk separately. In a recently completed population-based case–control study with a relatively large number of AA subjects, we examined whether RCC risk was elevated among participants with a history of cancer among first-degree relatives, and whether risk varied by race.

## Materials and methods

This population-based case–control study was conducted in Chicago, IL, and in Detroit, MI. Cases were resident Caucasian and AA men and women, aged 20–79 years, newly diagnosed with RCC in Chicago from 1 January 2003 through to 31 December 2003 and in Detroit from 1 February 2002 through to 31 January 2007 for AAs and through to 31 July 2006 for Caucasians. All cases had histologically confirmed adenocarcinoma of the kidney (ICD-O C64). Controls selected from the general population were frequency matched to cases on age, race, sex, and study centre. Controls aged 65–79 years were identified from files of the Centers for Medicare and Medicaid Services, and controls under the age of 65 years were identified from the Department of Motor Vehicle (DMV) records. A sampling strategy was designed to increase the number of AA participants used for analyses. All AA cases were recruited, whereas some strata (age–race–sex combinations) of Caucasian cases were subsampled. Controls were frequency matched to cases at a 2 : 1 ratio for AAs and at a 1 : 1 ratio for Caucasians. As information on race was unavailable from DMV records, this hampered our ability to frequency match controls to cases among those 20–64 years of age. Therefore, we used the racial density of the census block group (according to the 2000 census), in which each control resided to serve as a surrogate for race for the purposes of sampling. We oversampled people living in high-density AA areas to help achieve the targeted matching ratios for AAs.

Of 1918 eligible cases identified, 171 died before contact or interview, 92 could not be located with the available contact information, 21 moved out of the area, and the physicians of 63 cases refused permission to contact their patients. Among the remaining 1571 cases we sought to enrol, 221 declined participation and 133 were not interviewed because of serious illness, impairment, or failure to respond to multiple attempts to contact. Thus, 1217 cases (77.5% of those we attempted to recruit) participated in the study. Of 2718 presumed eligible controls, 41 died before contact or interview, 345 could not be located with the available contact information, and 63 had moved out of the region. Among the 2269 controls we attempted to recruit, 677 declined to participate and 357 were not interviewed because of serious illness, impairment, or failure to respond to multiple attempts to contact. Thus, 1235 eligible controls (54.4% of those we attempted to recruit) participated. Institutional review board approvals were obtained from all participating study centres and informed written consent was obtained from all participants.

Trained interviewers were administered an in-home, computer-assisted personal interview, in which detailed information was collected on demographics, smoking history, medical and medication history, diet, occupation, and family history of cancer. Information on family history of cancer was collected for all first-degree relatives (parent, sibling, and offspring), including cancer site and age at diagnosis. For analytical purposes, a set of sample weights were developed to reduce the potential for bias arising from differential sampling rates for controls and cases, from survey non-response, and from deficiencies in the coverage of the population at risk by the files of the DMV and Centers for Medicare and Medicaid Services to select controls. For controls, their sample weights also include a poststratification adjustment so that the weighted distribution of controls across the matching variables matches the weighted distribution of cases in an exact manner. In addition to being consistent with the objectives of frequency matching, this poststratification adjustment reduces the variability of the weights compared with not using this adjustment (Li *et al*, 2010).

The sample-weighted frequency distributions of selected characteristics and known RCC risk factors were compared between cases and controls using a Wald F-test. A *χ*^2^-test was also used to compare the unweighted sample distribution of selected characteristics in which similar findings were produced (data not shown). Unconditional logistic regression models using poststratified weights were used to calculate odds ratios (ORs) and 95% confidence intervals (95% CIs) associated with a family history of kidney cancer and a family history of non-renal cancers, using subjects who did not have any family history of cancer as the reference group. The jackknife replicate weight method was used to estimate s.e. (Rust and Rao, 1996). Stratified analyses by race were conducted, and interactions with race and family history were tested using a *t*-test and a Wald test. Regression models were adjusted for age at reference date (age at diagnosis for cases and age at study selection for controls), sex, race, study centre, education level, hypertension history (ever, never), family size (defined as number of siblings and number of offspring), smoking status, and body mass index. Unweighted unconditional logistic regression analyses and tests for interactions comparing regression models with and without interaction terms were also calculated, in which results were similar to that of the weighted analysis (data not shown). All analyses were conducted with STATA software version 10.1 (StatCorp, 2007). Statistical tests were determined to be significant at a two-sided *P*-value <0.05.

## Results

Overall, cases and controls were comparable in sex and age distributions (Table 1). As expected, cases were more likely to smoke, have hypertension, and have an excess body weight (body mass index ⩾30 kg m^−2^). Cases were more likely to have a lower education level than controls. Similar distributions of characteristics were observed when analyses were stratified by race.

A significant elevation in risk is shown in Table 2 among participants reporting at least one first-degree relative with kidney cancer (OR=2.29; 95% CI=1.31–4.00). Analysis by relative type revealed a non-significant increase in risk (OR=1.45; 95% CI=0.77–2.72) when the affected relative was a parent; however, a significant increase in risk was observed when a sibling was affected (OR=3.09; 95% CI=1.33–7.20). Stratified analyses showed no consistent differences in these associations by race, although the estimated risk associated with a parent having had kidney cancer was stronger among AAs (OR=2.98; 95% CI=1.06–8.37) than among Caucasians (OR=1.13; 95% CI=0.50–2.58) (*P*-interaction=0.19). A family history of non-renal cancers among first-degree relatives was not associated with RCC risk, regardless of whether family history was defined by relative type or stratified by race.

RCC risk was comparable whether one (OR=2.35; 95% CI=1.11–4.96) or more than one (OR=2.23; 95% CI=1.02–4.88) first-degree relative was reported to have been diagnosed with kidney cancer. The findings were similar when family history of non-renal cancers was reported in one (OR=0.93; 95% CI=0.75–1.14) compared with more than one (OR=1.09; 95% CI=0.80–1.49) first-degree relative. No meaningful differences in risk were observed with regard to relative type or race (data not shown).

Because the von Hippel–Lindau syndrome is commonly linked to inherited RCC, we examined the relationship between RCC risk and a family history of several cancers that have been reported in families with this syndrome (cancers of the retina, spinal cord, brain stem, cerebellum, adrenal gland, pancreas, broad ligament, and endolymphatic sac of the inner ear) (Kaelin, 2008). No association was observed (OR=0.99; 95% CI=0.74–1.32). A family history of smoking-related cancers (cancers of the lung, bladder, breast, cervix, colon, oesophagus, larynx, pancreas, stomach, oral cavity, and head and neck) was not related to RCC risk (OR=0.99; 95% CI=0.83–1.19). No statistically significant interactions between a family history of kidney cancer and potential confounders (i.e., body mass index, age, sex, smoking status, and hypertension history) were detected (data not shown). No meaningful difference in association was revealed when analyses were restricted to cases diagnosed with the clear-cell subtype of RCC or with high-grade (Furhman nuclear grades III and IV) tumours (data not shown).

## Discussion

This study confirms an association between RCC risk and a family history of kidney cancer and, for the first time, shows that such risk is comparable among Caucasians and AAs in the United States. Among Caucasians, the excess risk was observed mainly in individuals who reported having a sibling with kidney cancer; among AAs, excess risk was seen whether the affected relative was a sibling or a parent.

Increased risk of renal cancer has been associated with a family history of kidney cancer in most previous case–control (Mellemgaard *et al*, 1994; Schlehofer *et al*, 1996; Gago-Dominguez *et al*, 2001; Negri *et al*, 2006; Clague *et al*, 2009) and cohort studies (Goldgar *et al*, 1994; Czene and Hemminki, 2002; Gudbjartsson *et al*, 2002; Clague *et al*, 2009), although results for some are null or not statistically significant (McLaughlin *et al*, 1984; Kreiger *et al*, 1993; Hung *et al*, 2007; Randi *et al*, 2007). The reported excess risks generally ranged from two- to five-fold for both study designs. Most studies that have examined the type of first-degree relative with kidney cancer reported a stronger renal cancer risk if the affected relative was a sibling than a parent (Czene and Hemminki, 2002; Gudbjartsson *et al*, 2002; Negri *et al*, 2006; Hung *et al*, 2007). A recently published meta-analysis of seven case–control studies and three cohort studies reported an overall two-fold increase in RCC risk associated with a family history of kidney cancer, and the risk was nearly four-fold when the affected relative was a sibling (Clague *et al*, 2009). In this study, we also observed a greater risk with a reported history of kidney cancer in a sibling than in a parent, but this observation was confined to Caucasians. Because of the relatively small numbers of subjects with a family history of cancer, it is premature to conclude that there are racial differences in RCC risk by the type of first-degree relative affected with kidney cancer.

Elevated risk of sporadic RCC associated with a family history of kidney cancer may indicate an inherited component in aetiology or environmental exposures that are shared in families. Several genetic syndromes predisposing to familial RCC have been identified. The most common of the hereditary syndromes are germline mutations in the von Hippel–Lindau (*VHL*) tumour-suppressor gene on chromosome 3p, which is associated exclusively with the clear cell histological subtype (Czene and Hemminki, 2002; Hung *et al*, 2007; Chow and Devesa, 2008). In addition to RCC, individuals with this germline mutation are at an increased risk for developing tumours of the central nervous system, retina, endolymphatic sac of the inner ear, broad ligament, adrenal glands, and pancreas (Kaelin, 2008). However, we did not observe an association with a history of any of these *VHL-*related tumours among first-degree relatives. Shared environmental exposures among family members may also have a role in the associations observed in this study. We were unable to obtain exposure information for relatives of study participants or information on exposures during childhood; however, adjustment for known RCC risk factors, including smoking, obesity, and hypertension, did not modify the association between RCC risk and familial history of cancer. Until twin studies are conducted, it will be difficult to dissect the relative contributions of genetic factors and shared environmental exposures among family members on sporadic RCC risk. The largest published twin study has been uninformative on kidney cancer risk because of the lack of concordant twin pairs (Lichtenstein *et al*, 2000).

This is the first RCC case–control study to include a sufficient number of AAs to evaluate their risks separately. Histologically confirmed cancer and a large sample size are strengths, but power for stratified analysis was limited. The response rate among controls was not optimal (54.4%); however, the weighting strategies used in this study allowed for analyses that were more robust to non-response and the power for stratified analyses was limited. We did not verify the self-reported history of kidney cancer among first-degree relatives, and recall may have been more accurate among cases than among controls. However, we believe this to be unlikely as no association with an increasing number of affected first-degree relatives with kidney cancer was observed.

This study finds that family history of kidney cancer among first-degree relatives is associated with a significantly increased RCC risk, and that risk is similar in Caucasians and AAs.

## Figures and Tables

**Table 1:** Weighted characteristics of participants

	**All participants**					**Caucasian participants**					**African-American participants**				
	**Cases**		**Controls**			**Cases**		**Controls**			**Cases**	**Controls**			
**Variables**	***N***	**%^a^**	***N***	**%^a^**	***P*-value^b^**	***N***	**%^a^**	***N***	**%^a^**	***P*-value^b^**	***N***	**%^a^**	***N***	**%^a^**	***P*-value^b^**
Total	1217		1235			856		712			361		523		
															
*Sex*															
Males	720	61.8	689	61.4		495	62.0	439	61.6		225	61.3	250	60.8	
Females	497	38.2	546	38.6	0.10	361	38.0	273	38.4	0.07	136	38.8	273	39.2	0.54
															
*Age at reference date (years)*															
<45	147	10.5	179	10.5		106	10.2	93	10.2		41	11.6	86	11.6	
45–54	287	21.6	270	21.6		185	20.0	145	20.0		102	26.1	125	26.1	
55–64	372	29.4	350	29.4		255	29.1	205	29.1		117	30.2	145	30.2	
65–74	303	27.1	329	27.1		221	28.1	196	28.1		82	24.3	133	24.3	
75+	108	11.5	107	11.5	<0.99	89	12.7	73	12.7	<0.99	19	7.9	34	7.9	<0.99
															
Mean age (years)	59.9		59.9			60.5	60.4		58.5		58.4				
															
*Study center*															
Detroit	1018	83.3	1038	82.7		738	84.9	611	83.9		280	78.6	427	79.0	
Chicago	199	16.7	197	17.3	0.57	118	15.1	101	16.1	0.50	81	21.4	96	21.0	0.88
															
*BMI* c															
<25	240	19.5	366	29.1		172	19.6	216	29.4		68	19.1	150	28.1	
25–29.9	436	37.4	493	41.7		310	37.7	294	42.3		126	36.8	199	40.0	
30–34.99	298	24.9	221	18.3		210	25.2	126	18.1		88	23.9	95	18.9	
35+	230	18.2	147	10.9	<0.001	156	17.6	74	10.1	<0.001	74	20.2	73	13.1	0.01
															
*Smoking status*															
Never	432	35.3	471	38.4		309	35.8	287	39.9		123	33.9	184	34.3	
Occasional^d^	55	4.7	55	4.0		34	4.2	25	3.5		21	6.2	30	5.4	
Regular former smoker	410	34.7	445	38.0		304	36.5	276	39.3		106	29.4	169	34.1	
Regular current smoker	320	25.3	264	19.7	0.03	209	23.5	124	17.4	0.05	111	30.5	140	26.3	0.25
															
*History of hypertension*															
No	500	40.8	718	59.0		398	45.1	445	61.7		102	28.8	273	51.3	
Yes	701	59.2	508	41.0	<0.001	445	55.0	262	38.3	<0.001	256	71.2	246	48.7	<0.001
															
*Education level*															
<12 years	200	16.7	165	12.0		103	12.7	65	9.4		97	28.1	100	19.1	
High school graduate	419	34.5	390	31.5		315	36.6	214	30.8		104	28.7	176	33.5	
Some college	328	26.3	356	27.3		215	24.9	184	25.6		113	30.3	172	32.1	
College graduate	270	22.5	324	29.3	<0.001	223	25.9	249	34.2	0.001	47	12.8	75	15.3	0.01

Abbreviation: BMI=body mass index.

a A sample-weighted frequency distribution.

b *P*-value from sample Wald F-test.

c BMI 5 years before interview.

d Smoked 100 cigarettes in the lifetime, but never smoked at least 1 cigarette a day for 6 months or longer.

The following data are unknown: BMI (eight Caucasian cases, two Caucasian controls, five African-American cases, six African-American controls), history of hypertension (thirteen Caucasian cases, five Caucasian controls, three African-American cases, four African-American controls).

**Table 2:** Family history of cancer and risk of renal cell carcinoma, stratified by race

	**All participants^a^**						**Caucasian participants^b^**						**African-American participants^b^**						
	**Cases**		**Controls**				**Cases**		**Controls**				**Cases**		**Controls**				
	***N***	**%^c^**	***N***	**%^c^**	**OR**	**95% CI**	***N***	**%^c^**	***N***	**%^c^**	**OR**	**95% CI**	***N***	**%^c^**	***N***	**%^c^**	**OR**	**95% CI**	***P*-value^d^**
*Family history of kidney cancer*																			
*At least one first-degree relative with kidney cancer*																			
No	517	90.8	566	95.6	1.00		334	90.8	278	94.6	1.00		183	90.8	288	97.6	1.00		
Yes	52	9.2	24	4.4	2.29	(1.31–4.00)	33	9.2	15	5.4	1.98	(0.99–4.03)	19	9.2	9	2.4	3.96	(1.45–10.84)	0.34
																			
*At least one parent with kidney cancer*																			
No	517	94.2	566	95.9	1.00		334	94.6	278	95.0	1.00		183	93.4	288	97.9	1.00		
Yes	33	5.8	22	4.1	1.45	(0.77–2.72)	19	5.4	14	5.0	1.13	(0.50–2.58)	14	6.6	8	2.1	2.98	(1.06–8.37)	0.19
																			
*At least one sibling with kidney cancer*																			
No	517	94.3	566	98.1	1.00		334	94.0	278	98.1	1.00		183	95.0	288	98.2	1.00		
Yes	29	5.7	10	1.9	3.09	(1.33–7.20)	20	6.0	5	1.9	3.54	(1.18–10.62)	9	5.0	5	1.8	2.42	(0.61–9.60)	0.63
																			
*Family history of non-renal cancers*																			
*At least one first-degree relative with cancer*																			
No	517	43.9	566	43.1	1.00		334	40.2	278	38.9	1.00		183	54.6	288	55.0	1.00		
Yes	636	56.1	633	56.9	0.97	(0.79–1.19)	484	59.8	413	61.1	0.95	(0.75–1.21)	152	45.4	220	45.0	1.03	(0.76–1.41)	0.57
																			
*At least one parent with cancer*																			
No	517	51.1	566	50.0	1.00		334	46.7	278	45.2	1.00		183	63.9	288	63.2	1.00		
Yes	479	48.9	478	50.0	0.93	(0.75–1.15)	376	53.3	322	54.8	0.92	(0.71–1.18)	103	36.1	156	36.8	0.95	(0.68–1.32)	0.88
																			
*At least one sibling with cancer*																			
No	517	64.7	566	64.1	1.00		334	62.0	278	60.3	1.00		183	71.3	288	73.4	1.00		
Yes	269	35.3	265	35.9	0.98	(0.74–1.30)	195	38.0	166	39.7	0.93	(0.68–1.28)	74	28.7	99	26.6	1.16	(0.75–1.79)	0.36
																			
*At least one offspring with cancer*																			
No	517	94.6	566	93.7	1.00		334	92.7	278	91.8	1.00		183	99.0	288	97.7	1.00		
Yes	26	5.4	31	6.3	0.83	(0.47–1.48)	24	7.3	25	8.2	0.88	(0.48–1.62)	2	1.0	6	2.3	0.46	(0.04–5.77)	0.52

Abbreviations: CI=confidence interval; OR=odds ratio.

a Adjusted for age at reference date, sex, study center, smoking status, history of hypertension, body mass index, education, family size, and race.

b Adjusted for age at reference date, sex, study center, smoking status, history of hypertension, body mass index, education, and family size.

c A sample-weighted frequency distribution.

d Interaction *P*-value using a *t*-test.

The ‘No’ variable for all exposure–response groups represents participants with no familial history of cancer (or kidney cancer) among first-degree relatives.

Analysis for at least one offspring with kidney cancer was not calculated due to small numbers (four cases, one control).
